# Reducing Improper and Missed Peri-Procedure Antibiotics Prescriptions in a Regional Oncology Centre

**DOI:** 10.7759/cureus.59527

**Published:** 2024-05-02

**Authors:** Muhammad Khursheed Ullah Khan Marwat, Laila Laila

**Affiliations:** 1 Oncology, Hull University Teaching Hospitals NHS Trust, Hull, GBR; 2 Oncology, Hull University Teaching Hospitals NHS Trust, Cottingham, GBR

**Keywords:** oncology, oncology department, clinical governance, healthcare delivery system, quality improvement, clinical audit system, quality improvement projects

## Abstract

Background

In the realm of oncology care, patients undergoing invasive procedures are particularly vulnerable to infections due to their compromised immune systems. Antibiotics play a pivotal role in preventing such infections. However, inappropriate or missed administration of peri-procedure antibiotics poses a significant risk in the form of treatment complications, contributing to antibiotic resistance and increased healthcare costs.

Methods

The study was a two-cycle, closed-loop quality improvement project utilizing both retrospective and prospective data analysis of peri-procedure antibiotics prescription in a regional oncology centre. Two audit cycles were carried out in total; the first cycle was carried out in November 2023 where six-week data were collected retrospectively. As a result, formal and informal teaching sessions about the importance of correct peri-procedure antibiotics and the availability of complete institutional peri-procedure antibiotics guidelines in clinical areas were ensured. The second cycle was carried out prospectively for two weeks in January 2024. Patients were included if they underwent selected procedures performed by interventional radiology or gastroenterology while the patients operated on by the general surgeons and any day case procedures were excluded.

Results

We identified a total of 82 interventional procedures during the first cycle that fulfilled the inclusion criteria. Six out of 82 patients (7.3%) did not receive the correct peri-procedural antibiotics as per hospital antibiotics guidelines. A prospective two-week data after implementing the change revealed that 25 patients had documented interventional procedures done during this period using electronic patient records. Out of 25 patients, only one patient (4%) did not receive the peri-procedural antibiotics as per guidelines. We were able to demonstrate increased adherence to the peri-procedural guidelines (from 93% to 96%) during the two cycles. However, this change was not statistically significant (p = 0.50).

Conclusion

By educating and engaging healthcare professionals in adhering to evidence-based guidelines and best practices, we have observed notable, although statistically significant improvement in peri-procedure antibiotics prescription practices. Continued educational efforts and reinforcement strategies will be vital in further improvements over time. By providing ongoing support and resources, healthcare providers can be empowered to consistently make informed decisions regarding peri-procedure antibiotic administration. This commitment to maintaining high standards of antibiotic prescribing practices is expected to result in improved patient outcomes, including reduced rates of surgical site infections and antibiotic resistance. It is imperative to recognize the critical role that accurate peri-procedure antibiotic prescriptions play in patient safety and overall healthcare quality. By fostering a culture of continuous improvement and adherence to established guidelines, we can ensure that patients receive optimal care while minimizing the risks associated with antibiotic overuse or misuse.

## Introduction

Interventional radiology (IR) plays a significant role in the management of patients with cancer. Additionally, IR has taken on a significant role in the management of cancer complications, which can arise as a direct result of the disease or as a side effect of treatment. Various body systems and organs might malfunction because of cancer and many of these problems can be treated with less invasive IR procedures. A patient's quality of life can be greatly enhanced by this type of treatment, which can reduce or relieve pain, alleviate symptoms, and improve quality of life [1].

In the realm of oncology care, patients undergoing invasive procedures are particularly vulnerable to infections due to their compromised immune systems [2]. Antibiotics play a pivotal role in preventing such infections; however, the inappropriate or missed administration of peri-procedural antibiotics poses a significant risk, in the form of treatment complications, contributing to antibiotic resistance and increased healthcare costs [2-4]. Peri-procedure antibiotics play a critical role in preventing these infections by reducing bacterial burden in the surgical field and surrounding tissues. However, inappropriate antibiotic use, encompassing incorrect selection, dosage, or unnecessary prophylaxis, can contribute to treatment complications and the emergence of antibiotic resistance. Inappropriate use can also lead to missed opportunities to prevent potentially life-threatening infections [5].

Achieving optimal surgical antimicrobial prophylaxis (SAP) requires meeting the following critical quality indicators: appropriately choosing the antimicrobial for the indication, administering the dose via the appropriate route, giving intraoperative doses at the appropriate intervals, administering preoperative antibiotics at the appropriate time, and administering SAP for the recommended amount of time [6]. It has been proven that post-operative sepsis in endoscopic retrograde cholangiopancreatography (ERCP) is significantly reduced by peri-operative antibiotics administration [7]. Moreover, procedures such as percutaneous transhepatic cholangiography (PTC) have a high rate of infection [8]. Most of the patients having these procedures have advanced malignancy and may have underlying sepsis even before the procedure, and for this reason, the complication rate is high in patients with malignancy. The incidence of cholangitis in oncology patients undergoing PTC and drainage approaches 50% while the mortality rate is approximately 2%, with sepsis and haemorrhage being the two leading causes of death [9,10].

Studies have shown that several factors contribute to the issue of improper antibiotic prescription. These factors include variability in clinician knowledge regarding the ever-evolving landscape of antibiotic guidelines, lack of access to complete and standardized protocols at the point of care, and potential workflow inefficiencies that hinder timely and accurate prescribing [11].

This quality improvement project (QIP) aimed to address the challenge of improper and missed peri-procedural antibiotic prescriptions within oncology wards at Queen’s Centre for Oncology. Addressing this concern required a multifaceted approach encompassing interventions such as clinician education, implementation of evidence-based guidelines, and making the guidelines easily available to clinicians. By implementing targeted interventions, this QIP endeavoured to reduce the incidence of improper and missed peri-procedural antibiotic prescriptions, thus enhancing patient outcomes, minimizing antimicrobial resistance, and optimizing healthcare resource utilization.

## Materials and methods

Study setting and ethical approval

This study was conducted in a regional oncology centre affiliated with Hull University Teaching Hospital NHS Trust. The approval for the audit was granted by the Continuous Quality Improvement Department at Hull University Teaching Hospital NHS Trust. This quality improvement project (QIP) was conducted in the Queen’s Centre for Oncology, Cottingham, UK. This project did not meet the UK Health Research Authority criteria for research. Ethical approval was therefore not required, and the project was registered locally with the Continuous Quality Improvement Department (CQI) as a QIP.

Data analysis

The data were collected and analysed in Microsoft Excel (Microsoft Corporation, Redmond, WA), Microsoft Word, and SPSS (IBM Corp., Armonk, NY). The chi-squared test was applied to the prospective and retrospective data to assess the statistical significance of the change. The findings were then represented through figures, tables, and charts.

It was identified early on during the study that the hospital peri-procedure antibiotics guidelines missed important information like guidance about post-procedure antibiotics and alternate options in case of drug allergy to the recommended antibiotics. Therefore, infectious disease and interventional radiology departments were involved to revise and formulate comprehensive peri-procedure antibiotics guidelines for interventional procedures (Table 1). The revised guidelines now suggested alternatives if the recommended antibiotics could not be used, e.g., when patients have allergies to specific antibiotic groups.

**Table 1:** Institutional peri-procedure antibiotics guidelines PTC: percutaneous transhepatic cholangiography; ERCP: endoscopic retrograde cholangiopancreatography; RIG: radiologically inserted gastrostomy; US: ultrasound; CT: computed tomography; PICC line: peripherally inserted central catheter; IBW: ideal body weight; TDS: three times a day; STAT: immediately.

Procedure	Pre-procedure antibiotics	Post-procedure antibiotics
PTC + external drain	Co-amoxiclav 1.2 g + metronidazole 500 mg STAT; penicillin allergy: co-trimoxazole 960 mg + metronidazole 500 mg STAT	Nil
Biliary stent	Co-amoxiclav 1.2 G + metronidazole 500 mg STAT; penicillin allergy: co-trimoxazole 960 mg + metronidazole 500 mg STAT	Co-amoxiclav 1.2 g + metronidazole 500 mg TDS (continue for 48 hours post procedure); penicillin allergy: co-trimoxazole 960 mg + metronidazole 500 mg TDS STAT
ERCP	Gentamycin 5 mg/kg IBW (max 480 mg) STAT	Nil
Colonic stent	Co-amoxiclav 1.2 g + metronidazole 500 mg STAT; penicillin allergy: co-trimoxazole 960 mg + metronidazole 500 mg STAT	Nil
Oesophageal stent	Co-amoxiclav 1.2 g + metronidazole 500 mg STAT; penicillin allergy: co-trimoxazole 960 mg + metronidazole 500 mg STAT	Nil
Radiologically inserted gastrostomy (RIG)	Co-amoxiclav 1.2 g STAT; penicillin allergy: co-trimoxazole 960 mg + metronidazole 500 mg STAT	Nil
Nephrostomy	Gentamycin 5 mg/kg IBW (max 480 mg) STAT	Nil
Ureteric stent	Gentamycin 5 mg/kg IBW (max 480 mg) STAT	Nil
Nephrostomy exchange	Gentamycin 5 mg/kg IBW (max 480 mg) STAT	Nil
Ascitic drain	Nil	Nil
Chest drain	Nil	Nil
PleurX abdominal drain	Nil	Nil
US-guided biopsy	Nil	Nil
PICC line	Nil	Nil

Six-week data were collected retrospectively from electronic patient records (EPR) between 01-10-2023 and 12-11-2023, and the second cycle included two-week prospective data from 11-01-2024 to 25-01-2024. A list of commonly performed interventional procedures was compiled and only these listed procedures were included in the study (Table 2). Any day-case procedures or procedures not on the list were not included in the study. Electronic patient record for all the patients admitted during this period was screened to identify patients who had any procedures performed during admission. The appropriateness of peri-procedural antibiotic prophylaxis was then assessed based on revised institutional guidelines for antibiotic prophylaxis. A two-week prospective data about patients who underwent interventional procedures was then collected from 11-01-2024 to 25-01-2025 after the change was implemented.

**Table 2:** Procedures included in the study (inclusion criteria) PTC: percutaneous transhepatic cholangiography; ERCP: endoscopic retrograde cholangiopancreatography; CT: computed tomography.

S. No.	Procedure
1	PTC + external drain
2	Biliary stent
3	ERCP
4	Colonic stent
5	Duodenal stent
6	Oesophageal stent/dilatation
7	Radiologically inserted gastrostomy/exchange
8	Nephrostomy insertion/exchange
9	Ureteric stent
10	Ascitic drain
11	Chest drain
12	Ultrasound/CT-guided biopsy (liver/omental/lymph node/breast/bone)

Aim

The QIP aimed to achieve 100% adherence to the peri-procedure antibiotics prescription guidelines at the Queen’s Centre for Oncology.

Project design

After collecting and analysing data in cycle 1, through discussions with members of the multidisciplinary oncology team, we explored various strategies to improve prescribing practices. The primary focus was on developing a sustainable and multifaceted intervention that would effectively target the identified issues and emphasize the importance of appropriate antibiotic use for our oncology patients. The hospital’s continuous quality improvement (CQI) PDSA (Plan-Do-Study-Act) cycle design was used to identify the best change strategy to implement. A total of two PDSA cycles were completed to formulate the best possible interventions (Tables 3, 4). Various interventions were carried out, which included educating multidisciplinary team members about the importance of correct peri-procedure antibiotics prescription and pasting peri-procedure antibiotics guidelines posters in clinical areas. Qualitative data were collected from team members of the multi-disciplinary team about the usefulness of the new interventions using online surveys, and the majority of the feedback received from team members was positive.

**Table 3:** PDSA cycle 1 PDSA: Plan-Do-Study-Act; SMART: specific, measurable, achievable, relevant, and time-bound.

Aim (overall goal for this project) – What is your SMART aim?
100% adherence to peri-procedural antibiotics guidelines in oncology wards
PDSA cycle No.:
1
Change idea:
Educating junior doctors about the importance of correct peri-procedural antibiotics
PDSA objective: Describe the objective for this PDSA cycle
Increase awareness among the junior doctors and ultimately increase adherence to the peri-procedural guidelines
Predict what will happen when the test is carried out
Increased awareness among junior doctors about the correct peri-procedural antibiotic prescription
Measures to determine if prediction succeeds
Feedback from junior doctors

**Table 4:** PDSA cycle 2 PDSA: Plan-Do-Study-Act; SMART: specific, measurable, achievable, relevant, and time-bound.

Aim (overall goal for this project) – What is your SMART aim?
100% adherence to peri-procedural antibiotics guidelines in oncology wards
PDSA cycle No.:
2
Change idea:
Paste updated peri-procedural antibiotics guidelines in all oncology wards
PDSA objective: Describe the objective for this PDSA cycle
Availability of peri-procedural guidelines in all clinical areas
What questions do you want answered for this test of change?
Will the availability of complete guidelines increase adherence to them?
Predict what will happen when the test is carried out
Easy access to guidelines and less chance of improper or missed prescriptions due to lack of knowledge/resources
Measures to determine if prediction succeeds
Feedback from junior doctors and retrospective data of patients admitted between 11-01-2024 and 25-01-2024 to check adherence to the guidelines

## Results

Pre-change measurements

**Figure 1:**
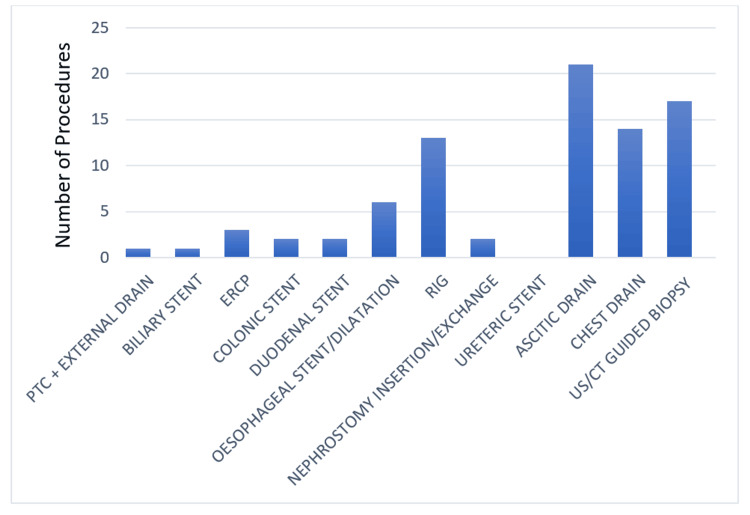
Number and type of procedures (pre-change) PTC: percutaneous transhepatic cholangiography; ERCP: endoscopic retrograde cholangiopancreatography; RIG: radiologically inserted gastrostomy; US: ultrasound; CT: computed tomography.

A total of 90 patients were identified during the first cycle; however, only 82 patients fulfilled the inclusion criteria. A total of 45 (54.8%) out of 82 patients were females while 37 patients (45.1%) were males. The most common procedure identified during the first cycle was abdominal ascitic drain insertion with 21 procedures (25.6%) identified while no cases of ureteric stent insertion were identified during the first cycle (Table 5 and Figure 1).

**Table 5:** Summary of the procedures performed between 01-10-2023 and 13-11-2023 PTC: percutaneous transhepatic cholangiography; ERCP: endoscopic retrograde cholangiopancreatography; US: ultrasound; CT: computed tomography.

S. No.	Procedure	Number performed
1	PTC + external drain	1
2	Biliary stent	1
3	ERCP	3
4	Colonic stent Insertion	2
5	Duodenal stent Insertion	2
6	Oesophageal stent insertion/dilatation	6
7	Radiologically inserted gastrostomy/exchange	13
8	Nephrostomy insertion/exchange	2
9	Ureteric stent Insertion	0
10	Abdominal ascitic drain insertion	21
11	Chest drain insertion	14
12	US/CT-guided biopsy (liver/omental/lymph node/breast/bone)	17
TOTAL		82

**Figure 2:**
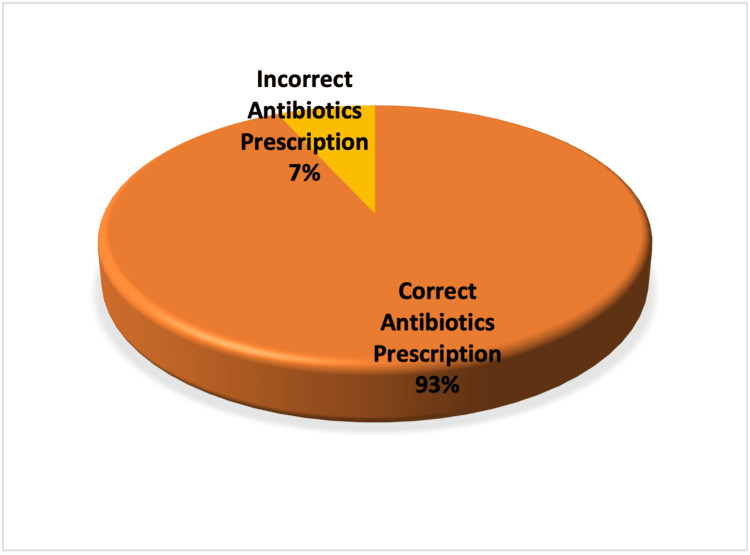
Adherence to antibiotics (pre-change)

It was found that six out of 82 patients (7.3%) either missed or did not receive the correct peri-procedure antibiotics as per guidelines, while the remaining 76 patients (92.7%) received the antibiotics as per guidelines (Figure 2). Among patients who did not receive correct antibiotics as per guidelines, two (33.3%) patients did not receive any antibiotics before their procedure, two (33.3%) patients received antibiotics that were not recommended by the guidelines, and the remaining two (33.3%) were prescribed post-procedure antibiotics while guidelines only suggested pre-procedure antibiotics. The procedures for which the antibiotics guidelines were not followed included colon stent insertion, radiologically inserted gastrostomy (RIG), ERCP, and PTC (Table 6). Several factors may contribute to these findings.

**Table 6:** Table identifying deviation from guidelines during cycle 1 PTC: percutaneous transhepatic cholangiography; ERCP: endoscopic retrograde cholangiopancreatography; RIG: radiologically inserted gastrostomy.

S. No.	Type of procedure	Deviation from guidelines
1	RIG	Given post-procedure antibiotics
2	ERCP	Missed pre-procedure antibiotics
3	PTC	Antibiotics not per guidelines
4	Colon stent	Missed pre-procedure antibiotics
5	ERCP	Antibiotics not per guidelines
6	RIG	Given post-procedure antibiotics

Post-change measurements

**Figure 3:**
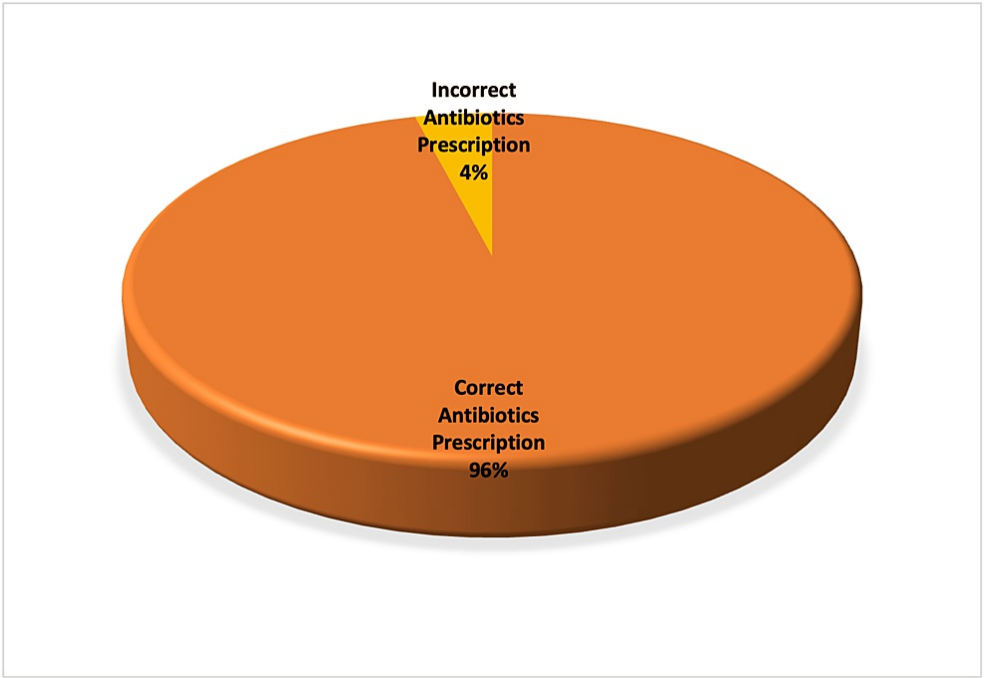
Adherence to antibiotics (post-change)

**Figure 4:**
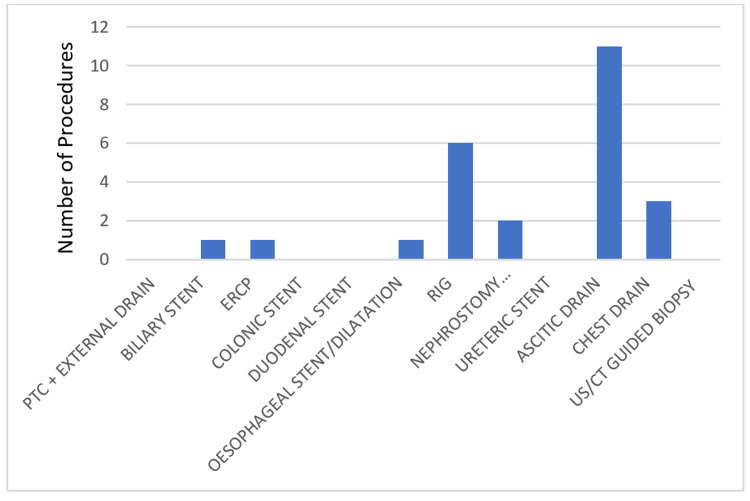
Number and type of procedures (post-change) PTC: percutaneous transhepatic cholangiography; ERCP: endoscopic retrograde cholangiopancreatography; RIG: radiologically inserted gastrostomy; US: ultrasound; CT: computed tomography.

During cycle 2, 33 patients were identified who had interventional and surgical procedures. However, only 75.7% (n = 25) of patients met the inclusion criteria. Of the patients, 48% (n = 12) were males while the remaining 52% (n = 13) patients were females (Table 7). Abdominal ascitic drain insertion was once again the most common procedure performed during cycle two comprising 44% (n = 11) of total procedures identified, followed by RIG comprising 24% (n = 6) of all cases identified while no PTC drainage, colonic/duodenal/ureteric stent insertion or ultrasound/CT-guided biopsy was identified during cycle 2 (Table 7 and Figure 3). Out of 25 patients, only one patient (4%) missed the pre-procedure antibiotics for ERCP. Of the identified patients, 96% (n = 24) received the correct peri-procedure antibiotics, as per institutional guidelines (Figure 4).

**Table 7:** Summary of the procedures performed (post-change) between 11-01-2024 and 25-01-2024 PTC: percutaneous transhepatic cholangiography; ERCP: endoscopic retrograde cholangiopancreatography; US: ultrasound; CT: computed tomography.

S. No.	Procedure	Number performed
1	PTC + external drain	0
2	Biliary stent	1
3	ERCP	1
4	Colonic stent insertion	0
5	Duodenal stent insertion	0
6	Oesophageal stent insertion/dilatation	1
7	Radiologically inserted gastrostomy/exchange	6
8	Nephrostomy insertion/exchange	2
9	Ureteric stent insertion	0
10	Abdominal ascitic drain insertion	11
11	Chest drain insertion	3
12	US/CT-guided biopsy (liver/omental/lymph node/breast/bone)	0
TOTAL		25

**Figure 5:**
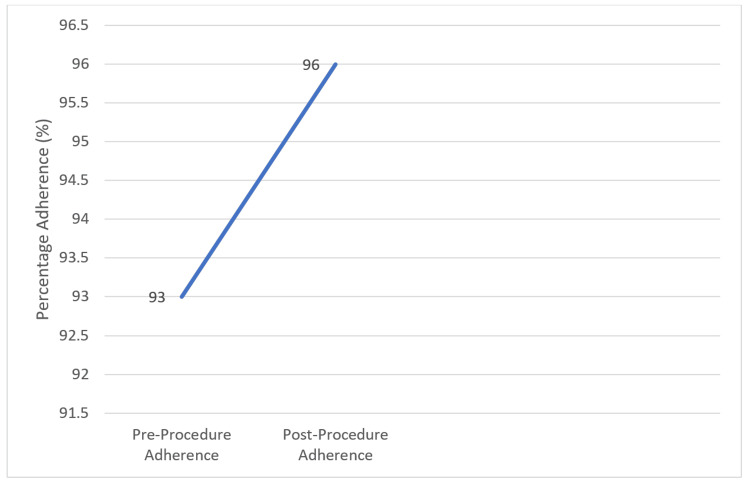
Peri-procedure antibiotics adherence improvement

A total of six instances of missed/incorrect peri-procedure antibiotic prescriptions were identified on electronic paper records (EPR) during cycle 1 of this retrospective-prospective study, which constituted 7% of the total procedures identified in cycle 1. After a change was implemented, a two-week prospective data revealed just one instance of non-compliance to the institutional peri-procedure antibiotics guidelines, which constituted 3% of the total procedures identified during cycle 2. This indicated an improvement of 4% in compliance with institutional peri-procedure antibiotics guidelines at 97%, which is up from 93% at baseline (p = 0.50) (Figure 5). Although this was not statistically significant, an increased awareness was observed among the members of the multidisciplinary team about the issue. It was, therefore, recommended that an ongoing audit of adherence to the peri-procedure antibiotics be carried out along with continuous education about this issue.

## Discussion

Our study identified a significant gap between current peri-procedural antibiotic prescribing practices and established institutional guidelines within our oncology wards. The initial audit revealed that 7% of patients did not receive appropriate antibiotic prophylaxis according to the guidelines. This highlights the potential for improvement in ensuring optimal antibiotic use for our immunocompromised patient population.

Several factors may contribute to these findings. A recent study suggests that variability in clinician knowledge regarding rapidly evolving antibiotic guidelines can be a significant barrier to adherence [12]. Additionally, the lack of readily accessible guidelines at the point of care and potential workflow inefficiencies that hinder timely and accurate prescribing have also been identified as contributing factors [13].

Following the initial audit, we implemented a multifaceted intervention to address these potential contributing factors. This intervention included educational sessions for relevant staff, focusing on the importance of appropriate antibiotic prophylaxis for specific procedures in patients. We also ensured the guidelines were readily available within the clinical areas and explored potential workflow modifications to streamline the prescribing process.

The re-audit data demonstrated a promising decrease in the rate of inappropriate prescribing to 4% after the intervention. This suggests that our multi-faceted approach may be effective in improving adherence to established guidelines. Our findings align with previous studies that have shown the effectiveness of educational interventions combined with improved access to guidelines in promoting appropriate antibiotic use [14].

Limitations

However, limitations exist. This study's retrospective design limits the ability to establish a causal relationship between the intervention and the observed improvement. Additionally, the relatively short follow-up period does not allow for an assessment of the intervention's long-term sustainability. Further research is warranted to explore these aspects. Future studies could include patient-reported outcomes and employ a prospective design to evaluate the intervention's effectiveness over a longer period. Additionally, qualitative research could be conducted to gain a deeper understanding of the specific factors influencing prescribing practices and identify potential areas for further improvement.

## Conclusions

Our quality improvement project demonstrated the potential for reducing inappropriate peri-procedural antibiotic use in oncology wards. The initial audit revealed a 93% rate of non-adherence to prescribing guidelines. Following the implementation of a multifaceted intervention, a re-audit showed a promising increase of 96%. This suggests that a collaborative approach involving education, guideline accessibility, and potential workflow modifications can improve prescribing practices and potentially contribute to better patient outcomes and reduced antimicrobial resistance. Further investigation is warranted to assess the long-term sustainability of these improvements.
